# Investigating shared risk variants and genetic etiology between Alzheimer’s disease and three stress-related psychiatric disorders: a large-scale genome-wide cross-trait analysis

**DOI:** 10.3389/fragi.2025.1488528

**Published:** 2025-02-05

**Authors:** Weijia Dang, Tianqi Hao, Ning Li, Hualin Zhang, Ziqi Li, Hongmei Yu, Yalu Wen, Deqiang Zheng, Long Liu

**Affiliations:** ^1^ Department of Health Statistics, School of Public Health, Shanxi Medical University, Taiyuan, Shanxi, China; ^2^ Department of Epidemiology and Health Statistics, School of Public Health, Capital Medical University, Beijing, China; ^3^ Department of Statistics, University of Auckland, Auckland, New Zealand; ^4^ Department of Health Statistics, School of Public Health, Binzhou Medical University, Yantai, Shandong, China

**Keywords:** Alzheimer’s disease, stress-related psychiatric disorders, genetic correlation, genome-wide cross-trait analysis, shared genetic etiology

## Abstract

**Introduction:**

Observational studies have reported that patients with Alzheimer’s disease (AD) have a greater burden of comorbidities typically associated with stress-related psychiatric disorders. However, the contribution of hereditary factors to this comorbidity remains unclear. We evaluated phenotypic associations using observational data from the UK Biobank.

**Method:**

Our study focused on investigating the shared risk variants and genetic etiology underlying AD and three stress-related psychiatric disorders: post-traumatic stress disorder, anxiety disorder, and major depressive disorder. By leveraging summary statistics from genome-wide association studies, we investigated global genetic correlations using linkage disequilibrium score regression, genetic covariance analysis, and high-definition likelihood. Genome-wide cross-trait analysis with association analysis based on subsets and cross-phenotype association were performed to discover genome-wide significant risk variants shared between AD and the three stress-related psychiatric disorders.

**Results:**

A significant positive genetic correlation was observed between AD and major depressive disorder using linkage disequilibrium score regression (rg = 0.231; *P* = 0.018), genetic covariance analysis (rg = 0.138; *P* < 0.001), and high-definition likelihood (rg = 0.188; *P* < 0.001). Association analysis based on subsets and cross-phenotype association revealed thirteen risk variants in six genes shared between AD and post-traumatic stress disorder; seven risk variants in four genes shared between AD and anxiety disorder; and 23 risk variants in four genes shared between AD and major depressive disorder. Functional annotation and gene-set enrichment analysis indicated that 12 genes for comorbidity shared between patients with AD and all three stress-related psychiatric disorders were enriched in the spleen, pancreas, and whole blood.

**Conclusion:**

These results advance our knowledge of the shared genetic origins of comorbidities and pave the way for advancements in the diagnosis, management, and prevention of stress-related AD.

## 1 Introduction

The classic features of Alzheimer’s disease (AD) include the accumulation of β-amyloid plaques and formation of neurofibrillary tangles containing hyperphosphorylated tau (Weller and Budson, 2018). Epidemiological evidence has revealed that stress-related psychiatric disorders may accelerate the onset of AD and worsen its course (Song et al., 2020; Rajkumar, 2023). Chronic stress increases the phosphorylation of tau and Aβ precursor proteins, which is linked to synaptic dysfunction and neuronal death in AD. It also activates the hypothalamic-pituitary-adrenal axis, which stimulates the production and secretion of stress hormones (Vyas et al., 2016; Sheng et al., 2021). Stress-related psychiatric conditions, known as post-traumatic stress disorder (PTSD), are characterized by the emergence of intrusive symptoms, avoidance of trauma-related cues, adverse changes in mood and cognition, and noticeable changes in arousal and reactivity after exposure to traumatic events (Merians et al., 2023). A recent epidemiological study reported that individuals with PTSD had a statistically significant propensity to develop AD (*HR* = 1.36; 95% *CI* = 1.12–1.67) (Song et al., 2020). Individuals with stress-related disorders have increased susceptibility to neurodegenerative diseases later in life, regardless of confounding factors such as environmental and familial influences. Along with PTSD, anxiety disorder (ANX) and major depressive disorder (MDD) are commonly referred to as “stress-related psychiatric disorders” (Smoller, 2016). An increase in anxiety has been correlated with elevated levels of β-amyloid, a protein linked to AD (Kwak et al., 2017), and the prevalence of ANX is between 9.4% (preclinical phase) and 39% (from mild to severe decline) in AD (Zhao et al., 2016; Bauer et al., 2018). Concomitant MDD is observed in 22%–59% of patients with AD (Starkstein et al., 2005), whereas the estimated lifetime prevalence of the general population is 11%–15% (Bromet et al., 2011). Clinical correlations between AD and MDD have been documented and are bolstered by their mutual impact on hippocampal shrinkage and participation of oxidative stress-related molecular pathways in the advancement of both conditions (Rodrigues et al., 2014). We first evaluated phenotypic associations using individual-level data from 255,896 participants from the UK Biobank (UKB). A reasonable hypothesis derived from neuropathological observational investigations is that AD is located on a continuum of stress-related psychiatric disorders, given the pathological and clinical overlap between AD and these illnesses (Guo et al., 2022). The recent exponential increase in the identification of risk variants affecting AD development has confirmed the role of genetic susceptibility (Nalls et al., 2019; van Rheenen et al., 2021; Wainberg et al., 2023). Comorbidities and genetic correlations between AD and stress-related mental health conditions suggest that both conditions share susceptibility variations, which frequently serve as genetic distorting factors in the relationships between traits. Next, we conducted a genome-wide cross-trait analysis to characterize the shared genetic architecture.

Given that hundreds of genetic variations influence many traits, the polygenic nature of complex traits leads to genetic variations that are shared across multiple phenotypes (Visscher et al., 2017). Genome-wide cross-trait analysis can better identify genetic variations in multiple traits or characteristics than single-trait research. This was performed using summary statistics from a large-scale genome-wide association study (GWAS) to infer a common genetic etiology (Zeggini and Ioannidis, 2009). We used association analysis based on subsets (ASSET) to identify shared genetic risk loci and conducted cross-phenotype association tests (CPASSOC) for the meta-analysis of associations across traits. ASSET, which is known for its ability to identify association signals across subsets of traits and accounts for potential sample overlap, complements CPASSOC by capturing single-nucleotide polymorphisms (SNPs) with opposite effects (Bhattacharjee et al., 2012). We used SNP-level functional annotation from Functional Mapping and Annotation (FUMA), to identify significant shared genomic risk loci based on ASSET and CPASSOC results. We used SNP enrichment to identify significant functional categories of different tissues involved in the comorbidity of AD and all three stress-related psychiatric disorders. In addition, we conducted the Kyoto Encyclopedia of Genes and Genomes (KEGG) pathway and cell-type-specific enrichment analyses with Web-based Cell-type-Specific Enrichment Analysis of Genes (WebCSEA) (Dai et al., 2022) to map genes with shared risk variants in their comorbidities.

Investigating the genetic etiology and shared risk variants of complex comorbidities is methodologically viable because of the availability of publicly available GWAS summary data and proven efficient methods. In this study, we investigated shared risk variants and biological pathways between AD and three stress-related psychiatric disorders (PTSD, ANX, and MDD). Genome-wide genetic correlation analyses and genome-wide cross-trait analysis will provide insight into the pathogenesis and therapeutics of AD with comorbidities.

## 2 Materials and methods

### 2.1 Observational analysis

The UKB study recruited 501,457 participants aged 37–73 years in the United Kingdom between 2006 and 2010 (Sudlow et al., 2015). Participants aged 60–70 years with available genotype information and White or British ethnic background were selected. The label “AD” was assigned to those in the UKB assessment center with one of the following ICD10 codes, relating to AD (G30.0, G30.1, G30.8, or G30.9). MDD, ANX, and PTSD were defined based on self-reported codes 1286, 1287, and 1469 (in the data field 20,002), respectively, with 199,125 participants remaining. Logistic regression models were used to determine whether AD was associated with MDD, ANX, and PTSD.

### 2.2 GWAS summary statistics and quality control

In this study, GWAS summary statistics for AD comprising 71,880 cases and 383,378 controls were obtained from https://www.ebi.ac.uk/gwas/studies/GCST007320 (Jansen et al., 2019). For PTSD, we downloaded the GWAS summary statistics of Nievergelt et al. (2019) from the Psychiatric Genomics Consortium (PGC), which included 23,212 cases and 151,447 controls. We obtained GWAS summary statistics for anxiety (ANX) from the PGC as reported by Otowa et al., which included 7,016 cases and 14,745 controls (Otowa et al., 2016). Summary statistics for patients with MDD, comprising 65,075 cases and 232,552 controls, were obtained from https://www.ebi.ac.uk/gwas/publications/34278373 (Glanville et al., 2021). During the quality control stage, we filtered out variants with minor allele frequency (MAF) less than 0.01, missing rate >5%, imputation r^2^ < 0.9, or those that deviated significantly from Hardy–Weinberg equilibrium (*P* < 1 × 10^−6^). Ambiguous SNPs (AT, TA, CG, and GC) were excluded from the analysis.

### 2.3 Investigation of shared genetics between stress-related psychiatric disorders and AD

**FIGURE 1:**
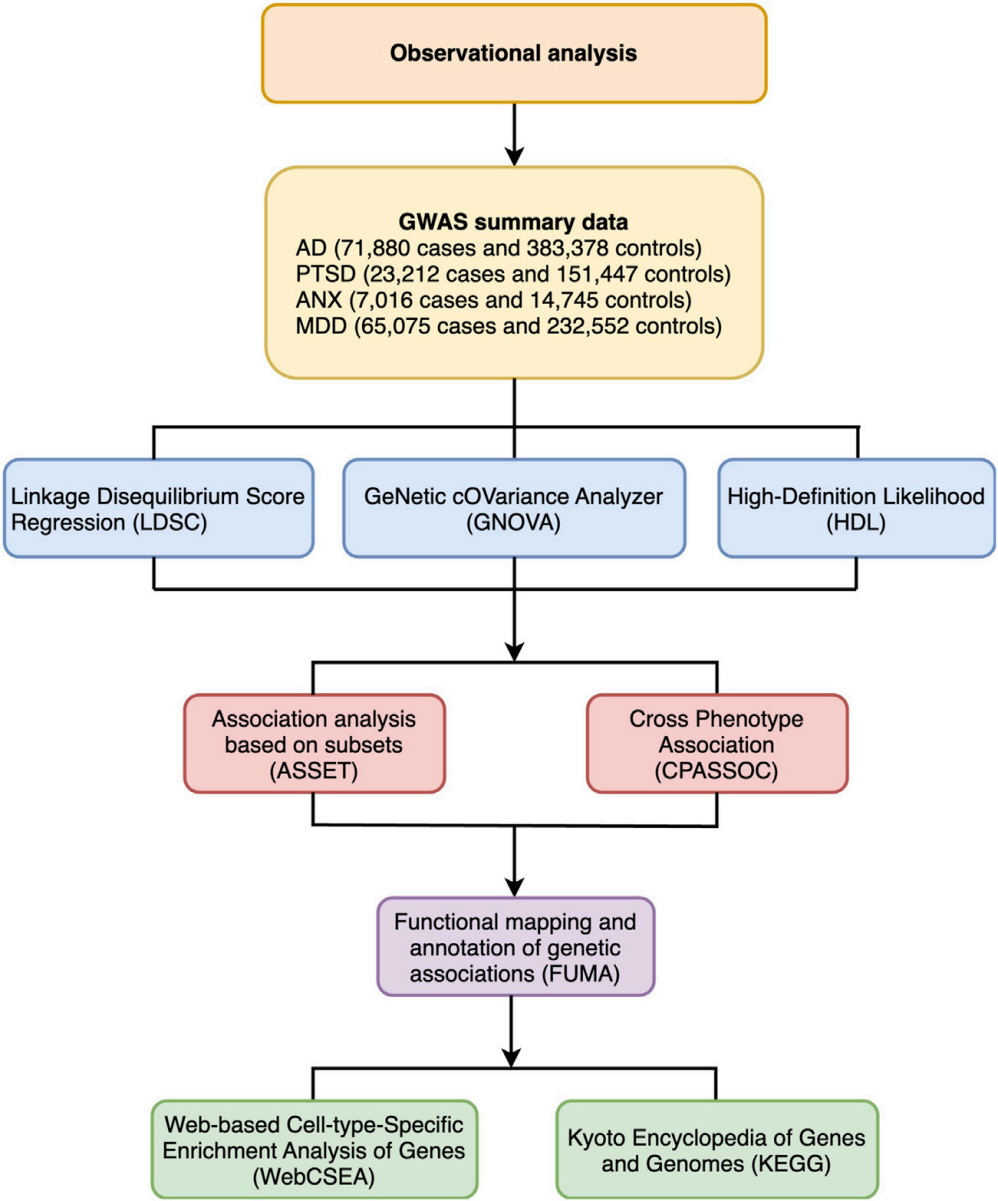
Flowchart of various statistical analyses for the present work. GWAS, Genome-wide association study; AD, Alzheimer’s disease; PTSD, Post-traumatic stress disorder; ANX, Anxiety disorders; MDD, Major depressive disorder; LDSC, Linkage disequilibrium score regression; GNOVA, Genetic covariance analyzer; HDL, High-definition likelihood; ASSET, Association analysis based on subsets; CPASSOC, Cross-phenotype association test; FUMA, Functional mapping and annotation of genetic association studies; KEGG, Kyoto Encyclopedia of Genes and Genomes; WebCSEA, Web-based Cell-type-Specific Enrichment Analysis of Genes.

To investigate shared genetics, we performed a genome-wide genetic correlation analysis, genome-wide cross-trait analysis, and functional annotation. The flowchart of the analysis is shown in Figure 1.

#### 2.3.1 Genome-wide genetic correlation analysis using LDSC, GNOVA, and HDL

Using the LDSC algorithm, which measures the average sharing of genetic influences across the entire genome between two phenotypes that are uninfluenced by environmental factors and confounders, we conducted a pairwise genetic correlation analysis (Bulik-Sullivan et al., 2015). Considering that linkage disequilibrium score regression (LDSC) only partially uses LD, we used genetic covariance analyzer (GNOVA) (Lu et al., 2017) and high-definition likelihood (HDL) (Ning et al., 2020) to gain a better understanding of the genetic correlation between AD and stress-related psychiatric disorders. For LDSC, we relied on the precomputed LD scores of the 1000 Genomes Project. After calculating the SNPs in the HapMap 3 SNP set, we reconstructed GWAS summary statistics and eliminated SNPs that did not match the reference panel (MAF ≤ 0.01 or INFO score ≤ 0.9) (Auton et al., 2015). LDSC analysis provides a means of quantifying the degree of genetic variation within populations without requiring individual variant genotyping. This method is most used to estimate the heritability and genetic correlations of complex traits. However, it is important to note that LD scores may not precisely capture genetic diversity in populations with diverse ancestries, potentially resulting in biased heritability and genetic risk score estimates. For GNOVA, we used the genetic correlation estimates with sample overlap correction (“corr_corrected” column from the GNOVA output) and the *P*-value for genetic covariance with sample overlap correction (“pvalue_corrected” column from the GNOVA output) in GNOVA (Wainberg et al., 2023). Compared with LDSC, GNOVA can quantitatively assess the contribution of different genetic variants to the phenotype and offers stronger statistical inference using genetic covariance. It can improve the estimated accuracy for genetic correlations, particularly in the case of moderate correlations, whereas LDSC makes only limited use of LD information. Furthermore, GNOVA and LDSC are robust methods for estimating genetic relationships as they are not affected by the sample overlap (Lu et al., 2017; Bulik-Sullivan et al., 2015). GNOVA incorporates information on sample overlap into its covariance estimation procedure, effectively controlling for this potential source of bias. For HDL, we estimated the genetic correlation using GWAS summary statistics, which decreased the variance of a genetic correlation estimate by approximately 60%, equivalent to a 2.5-fold increase in sample size (Ning et al., 2020). HDL can provide more precise model-fitting results by considering more data information, aiding in a better analysis of model uncertainty. It attempts to incorporate as much LD information as possible from the data, thereby addressing the limitations of the LDSC, which estimates genetic correlations based on partial LD information. However, the results may vary when different reference panels are used. Genetic correlations and causality between MDD and AD have been demonstrated in previous studies (Harerimana et al., 2022). In summary, we conducted genetic correlation analysis to assess pairwise global genetic correlations using LDSC, GNOVA, and HDL. These methods enhanced the statistical inference on comorbidities among AD and the three stress-related psychiatric disorders, thereby improving the precision of the genetic correlation estimation. In the GNOVA, the genetic covariance analysis included the Bonferroni correction to adjust the results. The LDSC utilizes the LD Score regression intercept to quantify the confounding bias.

#### 2.3.2 Genome-wide cross-trait analysis using ASSET and CPASSOC

Genetic correlation indicates that two qualities share genetic components, either because genetic variants influence one trait independently, or because genetic variants influence one trait through their influence on another (Shi et al., 2017). We used a subset-based meta-analysis method in ASSET to identify pleiotropic SNPs using all possible subsets of GWAS inputs (Li and Zhu, 2017). A *P*-value (multiple testing corrected) for the overall evidence of the association of a variant across phenotypes was returned by ASSET, with the best subset of phenotypes contributing to the overall association. ASSET searches all possible subsets of input GWAS traits for the greatest association signal, both positive and negative. Furthermore, as a sensitivity study, CPASSOC combines the association proof of GWAS summary statistics of several traits, where the variation is associated with at least one trait and controlled for population structure or cryptic relatedness. The CPASSOC results showed that pairwise *S*_*Het*_ that was calculated to combine summary statistics across traits and was an extension of *S*_*Hom*_, showed improved power with heterogeneous genetic effects (Zhu et al., 2015). We used the *S*_*Het*_ version for heterogeneous effects across traits. After the genome-wide cross-trait analysis, SNPs that reached genome-wide significance (*P*_ASSET_ < 5 × 10^−8^ and *P*_CPASSOC_ < 5 × 10^−8^) in paired traits were considered statistically significant shared risk variants. We focused only on the signals when the connection was influenced by many diseases.

#### 2.3.3 Genomic risk loci and functional annotation

Functional annotation of shared risk variants from ASSET and CPASSOC for AD and all three stress-related psychiatric disorders was performed using the FUMA web portal (Watanabe et al., 2017; Watanabe et al., 2019). Independent significant SNPs were identified as those that reached genome-wide significance (i.e., *P* ≤ 5.0 × 10^−8^) and were independent within a 1 Mb window (i.e., *r* ^*2*^ < 0.6). Lead SNPs were identified as several of the independent significant SNPs that had *r* ^*2*^ < 0.1 within a 1 Mb window. Genomic risk loci were identified using lead SNPs that were closer than a 250 kb distance. Subsequently, lead SNPs were mapped to the closest genes using ANNOVAR, and loci within 250 kb were combined into a single risk locus. For each locus, the top-lead SNP with the lowest *P*-value served as a representative. A novel shared risk gene was declared if it had never been reported in previous studies related to AD or any of the three stress-related psychiatric disorders.

SNP2GENE uses ANNOVAR to annotate SNPs based on the functional implications of gene function. Combined Annotation Dependent Depletion (CADD), potential regulatory functions (RegulomeDB), and chromatin states use FUMA. The risk of the SNPs, as indicated by 63 functional annotations, was reflected in the CADD score. We considered the most harmful variations as those with a CADD score of ≥12.37. The regulatory functionality of the SNPs, as shown by the RegulomeDB score, was derived from the overlap of the major difunctional data annotations available in the Genotype-Tissue Expression (GTEx) v8 dataset. The noncoding genome was annotated using ChromHMM, which predicts 15 categories based on 5 chromatin marks for 127 epigenomes, and FUMA shows the chromatin status access of genomic regions.

We used data from the GTEx v8 dataset provided by GENE2FUNC to create a heat map that visualized tissue-specific gene expression levels (e.g., brain, liver, and arteries) (Alemany et al., 2023). The predetermined differentially expressed gene sets in particular tissue types were obtained by comparing the normalized expression levels of each gene from one tissue with those of all other issues in the GTEx v8 dataset. We then used hypergeometric tests in GENE2FUNC to assess whether the mapped genes were overrepresented in the differentially expressed gene sets.

#### 2.3.4 KEGG pathway and cell-type-specific enrichment analysis

KEGG is an open and widely used database that integrates data on genomes, biological pathways, illnesses, and medications (Kanehisa and Goto, 2000). KEGG pathway analysis was performed to identify the pathways enriched with a list of significant proteins. The False Discovery Rate adjusted *P*-value on the pathway was computed to allow for multiple testing, where a value of <0.05 is regarded as significant.

WebCSEA is an online tool that provides a comprehensive exploration of the tissue-cell specificity of genes among the major human tissue-cell type maps (Dai et al., 2022). We used this tool to determine the cell-specific expression that may be involved in the pathogenesis of AD and all three stress-related psychiatric disorders for each mapped gene separately.

## 3 Results

### 3.1 Participant characteristics

Among the 501,457 participants in the UK Biobank, 4,130 individuals self-reported AD. In the current study, participants who self-reported AD were older and more likely to be female (81.62% of patients with AD compared to 47.42% of males). In the logistic regression models, people who reported AD were more likely to have depression (*OR* = 1.646, 95% *CI* = 1.433–1.880, *P* < 0.001) and ANX (*OR* = 1.332, 95% *CI* = 1.012–1.717, *P* = 0.033) (Table 1).

**TABLE 1:** Regression model for the association between AD and single-trait stress-related psychiatric disorders.

Trait	β	SE	*P*-value	OR	95%*CI*
Depression	0.498	0.069	<0.001	1.646	1.433–1.880
ANX	0.286	0.135	0.033	1.332	1.012–1.717
PTSD	−0.111	0.584	0.849	0.895	2.206–2.364

AD: Alzheimer’s disease, PTSD: Post-traumatic stress disorder, ANX: anxiety disorder.

### 3.2 Estimation of genetic correlations using LDSC, GNOVA, and HDL

The pairwise global genetic correlations between AD and stress-related psychiatric disorders are summarized in Table 2. Using LDSC, genetic correlations between AD and MDD, PTSD, and ANX were 0.231 (*P* = 0.018), 0.149 (*P* = 0.319), and 0.108 (*P* = 0.638), respectively. Using GNOVA, which allows the estimation of genetic correlations across continuous annotations, the genetic correlations between AD and MDD, PTSD, and ANX were 0.138 (*P* < 0.001), 0.074 (*P* = 0.154), and 0.104 (*P* = 0.188), respectively. Using the HDL method, which showed a significant positive genetic correlation, the genetic correlations between AD and MDD, PTSD, and ANX were 0.185 (*P* < 0.001), 0.128 (*P* = 0.072), and 0.457 (*P* = 0.411), respectively. The results of the three methods were largely consistent. AD and MDD showed significant positive genetic correlations, whereas no significant correlations were observed between AD and the other stress-related psychiatric disorders (PTSD and ANX).

**TABLE 2:** Pairwise genetic correlation between AD and single-trait stress-related psychiatric disorders.

Trait 1	Trait 2	LDSC			GNOVA			HDL		
		$r_{g}$	*SE*	*P*-value	$r_{g}$	*SE*	*P*-value	$r_{g}$	*SE*	*P*-value
AD	PTSD	0.149	0.149	0.319	0.074	0.003	0.154	0.128	0.071	0.072
	ANX	0.108	0.229	0.638	0.104	0.007	0.188	0.457	0.556	0.411
	MDD	0.231	0.097	0.018	0.138	0.003	<0.001	0.185	0.047	<0.001

AD: Alzheimer’s disease, PTSD: Post-traumatic stress disorder, ANX: anxiety disorder, MDD: major depressive disorder, LDSC: linkage disequilibrium score regression, GNOVA: genetic covariance analyzer, HDL: High-Definition Likelihood.

### 3.3 Genome-wide cross-trait analysis of AD and three single-trait stress-related psychiatric disorders

We identified 13 shared risk variants that reached genome-wide significance (*P*_ASSET_ < 5 × 10^−8^ and *P*_CPASSOC_ < 5 × 10^−8^) for AD and PTSD located within six genes (*APOC4-APOC2*, *APOE*, *TOMM40*, *CLPTM1*, *PVRL2*, and *CTB-179K24.3*) (Supplementary Table S1). The most significant SNP was rs1081105 (*P*_ASSET_ = 1.16 × 10^−231^, *P*_CPASSOC_ = 1.16 × 10^−232^), which is located within apolipoprotein E (*APOE*). It is a significant genetic risk factor for AD and increases the risk in homozygotes by up to 15 times in a dose-dependent manner (Strittmatter et al., 1993). The second most significant one was rs112019714 (*P*_ASSET_ = 1.72 × 10^−224^, *P*_CPASSOC_ = 2.02 × 10^−225^) that is found inside translocase of outer mitochondrial membrane 40 (*TOMM40*) and can activate the NOD-, LRR- and pyrin domain-containing protein 3 (*NLRP3*) inflammasome, microglia, and pro-inflammatory cytokines, which, in turn, can induce neurotoxicity in hippocampus neurons (Chen et al., 2023). Seven shared risk variants for AD and ANX, located within four genes (*BIN1*, *AP001257.1*, *PVRL2*, and *CASS4*), were identified (Supplementary Table S2). The most significant SNP was rs148303016 (*P*_ASSET_ = 6.26 × 10^−15^, *P*_CPASSOC_ = 3.96 × 10^−18^) located within poliovirus receptor-related 2 (*PVRL2*), followed by rs7575209 (*P*_ASSET_ = 3.69 × 10^−17^, *P*_CPASSOC_ = 1.66 × 10^−14^) located within bridging integrator-1 (*BIN1*), which is a major AD susceptibility gene (Lambert et al., 2022). Notably, *PVRL* expression, along with the AD GWAS-identified loci *TOMM40* and *APOE*, has been linked to the human lifespan. *PVRL* expression has been observed in various tissues, including the brain (Lu et al., 2014). We also identified 23 shared risk variants associated with AD and MDD (Supplementary Table S3) located in four genes (*BIN1*, *TMEM106B*, *PICALM*, and *SLC24A4*). The most significant SNP was rs1548884 (*P*_ASSET_ = 6.98 × 10^−16^, *P*_CPASSOC_ = 7.94 × 10^−16^) located in *TMEM106B*, a lysosomal transmembrane protein that has been closely associated with brain health (Zhang et al., 2023). Based on the results of three genome-wide cross-trait analysis with ASSET and CPASSOC, 12 shared genes (*APOC4-APOC2*, *APOE*, *TOMM40*, *CLPTM1*, *PVRL2*, *CTB-179K24.3*, *BIN1*, *AP001257.1*, *CASS4*, *TMEM106B*, *PICALM*, and *SLC24A4*) for comorbidity were identified for AD and all three stress-related psychiatric disorders, of which *AP001257.1* is a novel gene that has never been reported in previous AD-related or the three stress-related psychiatric disorders.

Twelve lead SNPs were located within 10 genes (*BIN1*, *TMEM106B*, *AP001257.1*, *PICALM*, *SLC24A4*, *PVRL2*, *APOE*, *CLPTM1*, *CTB-179K24.3*, and *CASS4*) for AD, and all three stress-related psychiatric disorders were identified using FUMA. In addition, six risk loci (2q14.3, 7p21.3, 11q14.2, 14q32.12, 19q13.32, and 20q13.31) were identified (Supplementary Table S4). The nearest gene to the most significant lead SNP rs1081105 (*P*_ASSET_ = 6.98 × 10^−16^, *P*_CPASSOC_ = 7.94 × 10^−16^) on the 19q13.32 risk locus was *APOE*. The gene-rich chromosome 19q13.32 has been linked to several adult human phenotypes, including lipid characteristics, AD, and longevity (Chiba-Falek et al., 2017). We also identified the three nearest genes (*PVRL2*, *APOE*, *CLPTM1*) that are all located in the 19q13.32 region. Two lead SNPs rs148303016 (*P*_ASSET_ = 6.26 × 10^−15^, *P*_CPASSOC_ = 3.96 × 10^−18^) and rs426555 (*P*_ASSET_ = 1.88 × 10^−23^, *P*_CPASSOC_ = 7.01 × 10^−24^) were all located within *PVRL2. BIN1* had two lead SNPs (rs7575209 and rs10200967) at locus 2q14.3, which is currently known as the most important genetic sensitivity locus in late-onset AD after *APOE* (Tan et al., 2014). The loci 7p21.3, 11q14.2, and 14q32.12 were mapped to transmembrane protein 106B (*TMEM106B*) (Satoh et al., 2014), phosphatidylinositol binding clathrin assembly protein (*PICALM*) (Ando et al., 2022), and sodium/potassium/calcium exchanger 4 (*SLC24A4*) (Yu et al., 2015) genes associated with AD, respectively. Patients with Parkinson’s disease have reduced *PICALM* mRNA expression in their blood, whereas patients with AD have higher levels. This study suggests that PICALM mRNA level in human blood could be a helpful diagnostic tool for distinguishing neurodegenerative illnesses from major depression (Kumon et al., 2021).

### 3.4 Functional annotation and gene set enrichment analysis results

**FIGURE 2:**
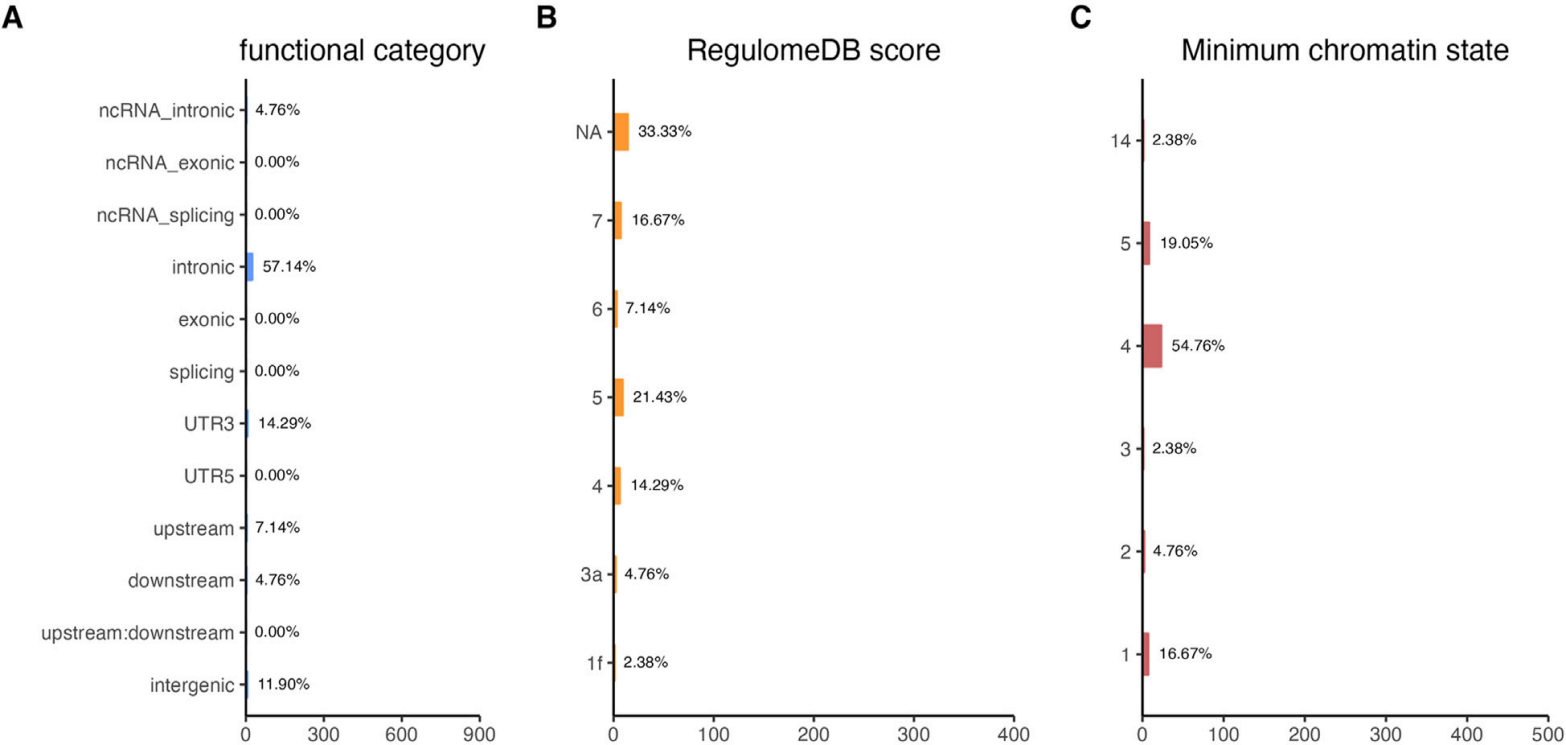
Distribution of the annotation for all SNPs jointly associated with the results of genome-wide cross-trait analysis among AD with three stress-related psychiatric disorders. **(A)** Distribution of functional categories of SNPs in the shared genomic risk loci. **(B)** Distribution of RegulomeDB score for SNPs in shared genomic loci. **(C)** The minimum chromatin state across 127 tissue and cell types for SNPs in shared genomic loci, with lower states indicating higher accessibility and states 1–7 referring to open chromatin states.

**FIGURE 3:**
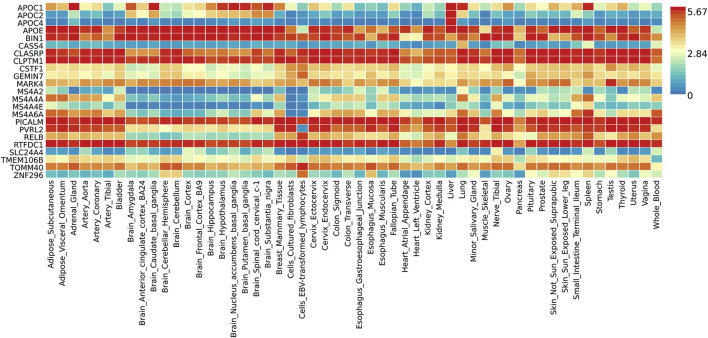
Shared genes expression heatmaps constructed with GTEx v8 (54 tissues). Genes and tissues are ordered by clusters for the GTEx heatmap. The abscissa represents the GTEx v8 tissues and the ordinate represents the genes selected by ASSET and CPASSOC.

**FIGURE 4:**
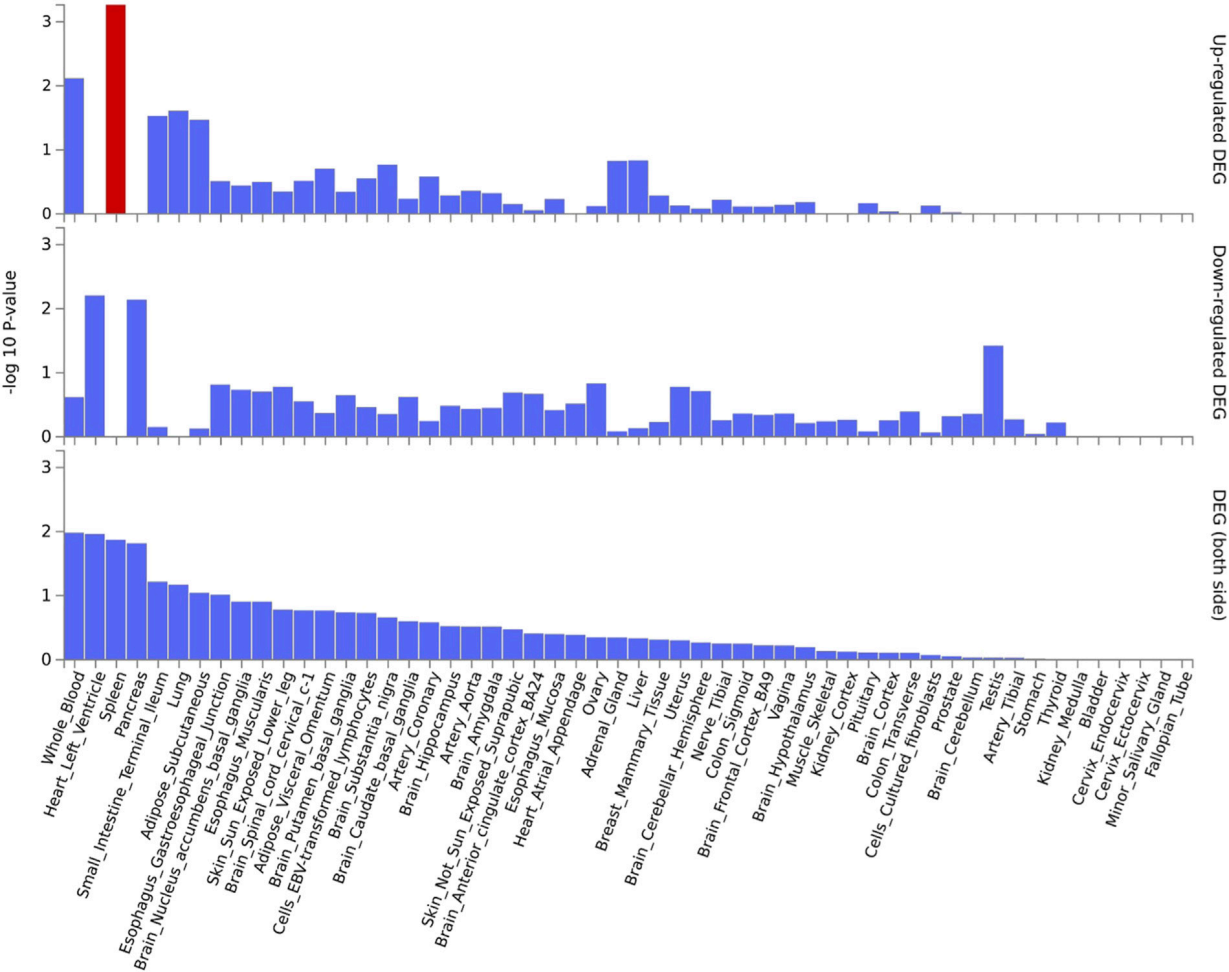
GTEx tissue enrichment analysis. Red bar represents significant tissue enrichment after Benjamin-Hochberg correction. The abscissa represents the GTEx v8 tissues and the ordinate represents the genes selected by ASSET and CPASSOC.

Functional annotation analysis of all the shared risk variants selected based on the genome-wide cross-trait analysis of AD and the three stress-related psychiatric disorders showed that the SNPs were mostly intronic and intergenic. Functional annotation revealed the over-presentation of SNPs in introns (57.1%), intergenic regions (11.9%), and non-intronic RNA (4.76%) (Figure 2A). A total of 97.62% of the variants within credible sets were in open chromatin regions (minimum chromatin state ≤ 7), 2.38% were likely to affect the binding of transcription factors (RegulomeDB scores from 1b to 2c), and 6.52% may be deleterious (CADD score > 12.37). According to the RegulomeDB score categories 1a–1f, variations are likely to have an impact on binding and are connected to the target gene’s expression. RegulomeDB ratings for AD ranged from 1a to 1f for 2.38% of SNPs (Figure 2B). Interestingly, the highest-scoring SNP (rs1081105) in the AD study had a RegulomeDB score of 5. A total of 97.62% of the candidate SNPs across AD and the three stress-related psychiatric diseases were in open chromatin state regions according to the minimal chromatin state distribution (Figure 2C). The tissue-specific gene expression levels of the genes that were co-housed using eQTL mapping of the common SNPs and found in our genome-wide cross-trait analysis are displayed in Figure 3. Notably, a genome-wide cross-trait analysis revealed that six genes (*APOE*, *BIN*, *CLASRP*, *CLPTM1*, *PICALM*, and *RTFDC1*) were highly expressed across all tissue types. As shown in Figure 4, genes that were mapped using eQTL using significant SNPs identified by genome-wide cross-trait analysis were enriched in the spleen, pancreas, and whole blood.

### 3.5 KEGG pathway and cell type specificity results

**FIGURE 5:**
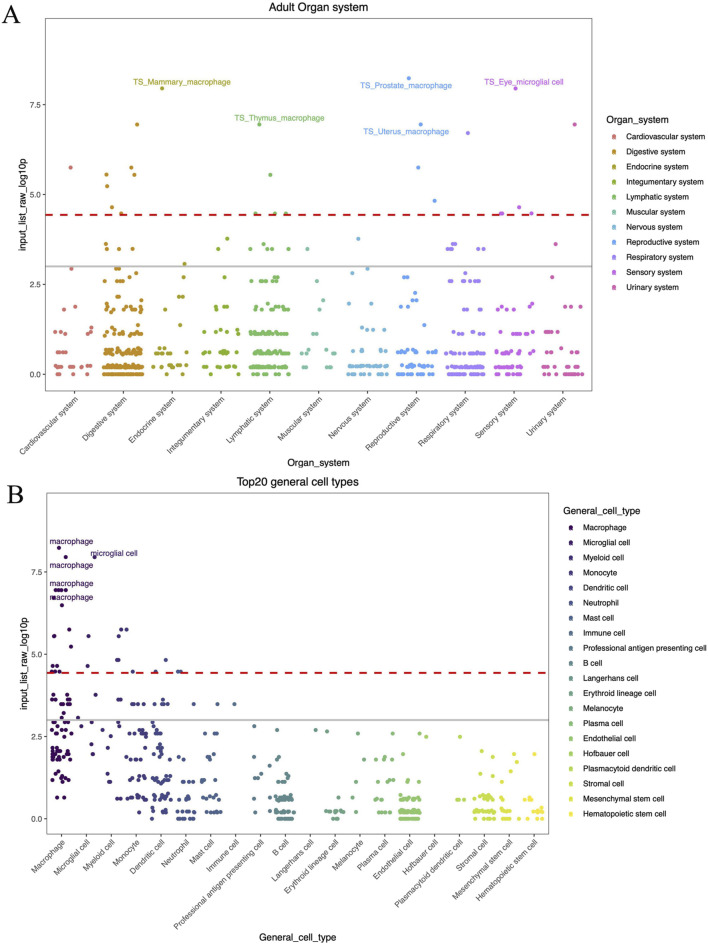
**(A)** WebCSEA top enriched organ systems. **(B)** WebCSEA top enriched celltypes. The red dashed line indicates the Bonferroni-corrected significance (*P* = 3.69 × 10^−5^) by 1,355 TCs. The grey solid line indicates the nominal significance (*P* = 0.001). The *X*-axis represents the components in different stratification strategies. *Y*-axis indicates the (−log 10 (combined *P*-value)) for each tissue-cell type from cell-type specificity enrichment analysis result.

We employed Enrichr to enhance the common risk genes in a KEGG functional analysis to understand the influence of risk genes on biological pathways. Two biochemical pathways were substantially enriched in AD, namely cholesterol metabolism and Fc gamma R-mediated phagocytosis. Notably, the strongest enrichment signal for AD and the three stress-related psychiatric disorders was for cholesterol metabolism, which included one enriched gene (*APOE*). The second enriched signal was for Fc gamma R-mediated phagocytosis, which included one enriched gene (*B1N1*). Finding the Fc gamma receptor-mediated phagocytosis pathway links the pathogenesis of AD to the peripheral innate immune system (Park et al., 2020). Furthermore, we found an enrichment of the mapped genes selected by genome-wide cross-trait analysis in the endocrine, lymphatic, reproductive, and sensory systems (Figure 5). The immune and nervous system-related macrophages and microglial cell types were the most enriched. These cells are essential for maintaining immunological homeostasis in a constant state and healing the tissue damage sustained during brain development or disease-related pathologies (Amann et al., 2023).

## 4 Discussion

In this study, we conducted a comprehensive observational and genetic analysis.

In the observational analysis, we used data from the UK Biobank to explore the associations between AD and stress-related psychiatric disorders in the population. Our observational analysis revealed links between AD and depression and between AD and ANX. We then conducted genetic analysis to systematically investigate the phenotypic associations, genetic correlations, pleiotropic loci, and gene expression between AD and three stress-related psychiatric disorders. In this genome-wide cross-trait analysis, we confirmed the risk-increasing relationship between AD and MDD, providing insights into the underlying biological mechanisms. Although LDSC analysis revealed no significant genetic correlation between AD and PTSD or ANX, this may be attributed to noise in the PTSD and ANX GWAS datasets or the inherently low genetic correlation with AD. To further explore their comorbid relationships, we assessed pairwise genetic correlations between traits to uncover the common etiologies underlying AD and stress-related psychiatric disorders. A genome-wide cross-trait analysis was used to identify pleiotropic genomic SNPs and genes shared between the two comorbidities, providing new perspectives on the potential biological mechanisms underlying these diseases. The findings of this study indicate that complementary approaches such as ASSET and CPASSOC are powerful methods for identifying shared risk variants in well-established comorbidities. It is crucial to elucidate the pleiotropic effects of key variations that support a high degree of comorbidity. Our findings are reliable because the ASSET-identified SNPs were also significant in CPASSOC analysis, as demonstrated by our results. ASSET leverages significant associations within specific subsets to aid the discovery of genetic variations across different populations. ASSET provides a better interpretation of comorbidity outcomes in case-control studies and enhances the capability to detect specific variants. However, the computational burden when handling summary data is substantial. CPASSOC can reveal underlying shared genetic bases, aiding in the discovery of genetic correlations between phenotypes. While compared to ASSET, it enhances the statistical power, it focuses more on the analysis of pleiotropy. Additionally, we functionally characterized shared risk genes using an integrative functional annotation platform that offers a wealth of information on variant and indel functional annotations. Our study revealed shared genetic variants or loci that likely contribute to the co-occurrence of AD with stress-related psychiatric disorders in European ancestry populations and aimed to obtain a deeper understanding of the molecular biological mechanisms that are essential to the onset and course of AD.

Genetic correlation provides valuable insights into the polygenic genetic architecture of complex phenotypes by quantifying overall genetic similarity (Anttila et al., 2018). Recent studies have demonstrated the superior accuracy of other methods, such as the GNOVA and HDL in simulations (Zhang et al., 2021b). Given the increase in publicly accessible GWAS summary data and the availability of efficient analytical tools, multi-trait joint analyses have become methodologically viable. According to the simulated experiments, no strategy based on summary data was effective in estimating genetic associations when the reference panel was mismatched (Zhang et al., 2021b). As the number of common SNPs between the reference panel and the GWAS decreased, the performance of the HDL declined. Therefore, a suitable reference panel is essential when using summary statistics-based approaches. Previous research has not identified genetic associations between AD and stress-related psychiatric diseases; however, using LDSC, GNOVA, and HDL, we found a strong positive correlation between the genomic architecture of AD and MDD. The genetic correlations calculated using GNOVA and HDL were consistent with those obtained using LDSC and explained the stability of our results. One possible explanation for this finding is the risk variants shared between AD and MDD. By implementing a genome-wide cross-trait analysis, we discovered shared genes that have not been previously reported in other comorbidity genetic studies on AD.

In the large-scale genome-wide cross-trait analysis, we found that 12 genes were mapped from 43 shared risk variants in three cross-trait groups (AD and PTSD, AD and ANX, and AD and MDD) using FUMA. Paired immunoglobin like type 2 receptor alpha (PILRA) has long been recognized as a risk gene for AD; alongside other functional genes that participate in neuroinflammation are putative or proven calmodulin-binding proteins for other neurodegenerative diseases (O’Day and Huber, 2022). Human data are beginning to emerge suggesting that those who experience prolonged stress during their early years have a higher risk of developing AD later in life. Mitochondrial dysfunction is observed around senile plaques, notable lesions constituting aggregated Aβ and tau protein; *TOMM40* is implicated in the inflow of proteins and Aβ into mitochondria (Wang et al., 2022). Recent research has demonstrated the critical roles of non-coding RNAs in pathophysiological processes, including tau phosphorylation, oxidative stress, Aβ aggregation, cell proliferation and death, neuroinflammation, and autophagy, thus contributing to AD (Zhang et al., 2021c). Whether the long non-coding RNA *AP001257.1* is involved in the pathophysiology of AD requires further study. *APOE4* is associated with an earlier onset and a higher risk of AD. *APOE2* appears to increase the prevalence and severity of PTSD, although it is protective against AD (Johnson et al., 2015). However, biochemical mechanisms underlying this association remain unknown. In this study, we identified common genes between PTSD and AD. The *APOE ε4* allele has been found to be associated with the overactivation of microglia, which may exacerbate neuroinflammation and promote AD progression. In contrast, *APOE ε2* is thought to have stronger anti-inflammatory properties, which might help alleviate amyloid accumulation and reduce neuroinflammation. In the context of psychiatric disorders (such as depression, schizophrenia), *APOE* may also influence disease development and progression by regulating immune responses and inflammation. Although the exact mechanisms in psychiatric diseases are still not fully understood, some studies suggest that the *APOE ε4* allele is associated with increased neuroinflammation and macrophage activation, which could lead to immune system imbalance, potentially serving as a risk factor for certain psychiatric disorders.

Although a consensus has emerged from observational studies on AD and stress-related psychiatric disorders, the shared genetic etiology as a pathogenic mechanism remains unclear (Protsenko et al., 2023). *APOE* on 19q13.32, *APOC4-APOC2* on 19q13.32, and *TOMM40* on 19q13.32 were previously shown to be associated with AD and cognitive impairment (Cruz-Sanabria et al., 2021). Furthermore, we prioritized candidate genes from FUMA through genome-wide cross-trait analysis and inferred the biological pathways identified through functional annotation analyses. KEGG pathway analysis showed that the candidate genes were often part of biological pathways involving Fc gamma receptor-mediated phagocytosis in AD. Currently, there is no evidence linking stress-related psychiatric disorders to the common genetic architecture of AD, highlighting the complexity and heterogeneity of neurodegeneration and neuroinflammation as distinct processes with multiple paths (Stolp Andersen et al., 2022). KEGG provides extensive gene function and pathway information, covering multiple biological processes and signaling pathways. However, it does not consider variations across different conditions or tissues, which may limit its ability to explain changes in gene functions and pathways in different biological contexts. Functional and genetic annotation results suggest that the genes mapped from the genome-wide cross-trait analysis-identified SNPs have different functions in AD.

A lower RegulomeDB score indicated a higher probability of a regulatory role. Furthermore, a range of tissues, including the pancreas, spleen, and whole blood, were shown to have high enrichment levels of shared genes in a genome-wide cross-trait analysis. The spleen is a key organ of the immune system, playing a critical role in immune responses throughout the body via the blood. In neurological research, the spleen is believed to potentially influence the central nervous system immune environment. Immune factors released by the spleen may contribute to neuroinflammation, particularly in the pathology of AD and multiple sclerosis. The pancreas is not only involved in glucose metabolism but also communicates with the nervous system through the secretion of hormones like insulin and glucagon. These hormones regulate brain metabolic states and neuronal functions, particularly in mood regulation, cognition, and sleep. These tissues play important roles in regulating hormone and enzyme functions. The immune and nervous system microglia and astrocytes, which are notably enriched cell types, play pivotal roles in AD pathogenesis. Therefore, targeting microglia and astrocytes may offer a novel therapeutic approach for AD treatment. Inflammation, which is recognized as a significant trigger of AD onset, can precede amyloid deposition and contribute substantially to AD pathology (Heneka et al., 2013). Notably, Aβ deposits, chronic microglial activation, and microglial inflammatory mediators are pivotal in fueling the inflammatory cascade in AD progression (Zhang G. et al., 2021). Moreover, psychopathologies, particularly MDD, have been associated with the sustained priming and sensitization of cerebral microglia. Recent evidence suggests that the altered morphology and function of microglia induced by intense inflammatory activation or senescence may contribute to major depression and the associated impairments in neuroplasticity and neurogenesis (Rahimian et al., 2021).

Under normal circumstances, cholesterol metabolism is crucial for many cellular processes, such as hormone synthesis, serving as an energy source, and functioning as a component of the plasma membrane. Anxiety and depression are among the many pathogenic illnesses caused by dysregulated cholesterol metabolism, which also causes other neuropsychiatric disorders. Patients with neuropsychiatric illnesses also experience problems in cholesterol metabolism. Consequently, there is a strong correlation between metabolic abnormalities and neuropsychiatric illnesses. The dysregulation of cholesterol metabolism has also been observed in individuals with neuropsychiatric disorders. The pathogenesis of these disorders may involve immunological disruption, neuroinflammation, oxidative stress, and dysregulation of the neurotransmitter system. Individuals with neuropsychiatric disorders are expected to have a higher likelihood of developing metabolic disorders, such as metabolic syndrome. This disease is characterized by abnormalities in neuronal homeostasis, including toxicity and death of neurons, as well as changes in the functions and structures of neurons, including axonogenesis, synaptogenesis, neurogenesis, and action potentials, all of which are affected by cholesterol. Therefore, restoring aberrant or impaired cholesterol metabolism may aid in repairing the neuronal damage associated with neuropsychiatric disorders. A study by Feringa and Kant explored the connection between impaired cholesterol metabolism and neuropsychiatric illnesses and explains how aberrant cholesterol metabolism in neuropsychiatric disorders causes neuronal dysfunction (Feringa and van der Kant, 2021). Dysregulated cholesterol metabolism, which is implicated in neuropsychiatric disorders, presents opportunities for novel therapeutic interventions, potentially benefiting both stress-related illnesses and AD. Given the interconnectedness between metabolic abnormalities and neuropsychiatric conditions, addressing aberrant cholesterol metabolism could help alleviate the neuronal dysfunction associated with neuropsychiatric disorders. This underscores the importance of understanding the links between impaired cholesterol metabolism and neuropsychiatric disorders and offers insights for targeting new pathways in drug therapy for these conditions (Caruso et al., 2019). Cholesterol metabolism significantly affects macrophage and microglia activation. Both excessive and deficient cholesterol levels can alter immune cell function, leading to inflammation. In the brain, this dysregulation is particularly important for such as AD, where microglial activation and cholesterol metabolism are closely intertwined. Modulating cholesterol metabolism may offer therapeutic potential for diseases where inflammation plays a critical role.

This study had a few limitations. First, the generalizability of our findings was confined to populations of European ancestry because of the lack of available GWASs in non-European populations. Further investigations are needed to explore the shared genetic etiology between AD and stress-related psychiatric disorders in populations of other ancestries. Second, the genetic connections of rare variants could not be assessed because SNPs with MAF < 0.01 were automatically filtered in genetic correlation and genome-wide cross-trait analysis. Although we used a large sample population whenever possible, due to limitations in the disease data, we were unable to consider the impact of rare variants and population stratification on the results. This is an inherent limitation of GWAS studies. Additionally, cross-trait analysis is not feasible for GWAS summary statistics with small sample sizes or low SNP heritability. In the current study, for instance, the sample size for ANX was modest. Finally, we did not investigate the functional implications of the common risk loci that underlie our results, which are necessary to validate the molecular pathways in subsequent studies.

## 5 Conclusion

In conclusion, we provide new insights into the common genetic etiology between AD and three stress-related psychiatric diseases by using the largest genome-wide genetic dataset available to date and sophisticated statistical genetic techniques. Strong genetic correlations between AD and MDD were identified using genetic correlation estimates. A genome-wide cross-trait analysis revealed shared risk variants and genes associated with AD and stress-related psychiatric disorders. We identified 12 shared risk genes, including a novel long non-coding RNA gene that has not been previously reported in AD or any of the three stress-related psychiatric disorders. Our findings provide a new understanding of the pathogenic mechanism underlying the regulatory roles of AD and require further investigation at the molecular level.

## Data Availability

The data analyzed in this study is subject to the following licenses/restrictions: This research was conducted using resources from the UK Biobank under approved application number 95259. The GWAS summary statistics data that were examined were acquired from worldwide research consortia and public repositories. Requests to access these datasets should be directed to https://www.ukbiobank.ac.uk and https://www.ebi.ac.uk/gwas/home.

## Abbreviations

AD, Alzheimer’s disease; PTSD, Post-traumatic stress disorder; ANX, Anxiety disorder; MDD, Major depressive disorder; SNPs, Single nucleotide polymorphisms; GWAS, Genome wide association studies; PGC, Psychiatric Genomics Consortium; LDSC, Linkage disequilibrium score regression; GNOVA, Genetic covariance analyzer; HDL, High-Definition Likelihood; ASSET, Association analysis based on subsets; CPASSOC, Cross-phenotype association test; GTEx, Genotype-Tissue Expression; FUMA, Functional mapping and annotation of genome-wide association studies; KEGG, Kyoto Encyclopedia of Genes and Genomes; WebCSEA, Web-based Cell-type-Specific Enrichment Analysis of Genes.
