# Supplementary material for: Investigating shared risk variants and genetic etiology between Alzheimer’s disease and three stress-related psychiatric disorders: a large-scale genome-wide cross-trait analysis
Source: Front Aging. 2025 Feb 5;6:1488528. doi: 10.3389/fragi.2025.1488528 (PMC11837265; doi:10.3389/fragi.2025.1488528)
Supplement: Supplementary file 1 [file Table1.docx]

Supplementary Material

# Supplementary Tables

| **SNP** | **chromosome** | **position** | **Chromosmoe**  **Loci** | **A1** | **A2** | ***P_CPASSOC_*** | ***P_ASSET_*** | **DIST** | **Genes within clumping region** |
| --- | --- | --- | --- | --- | --- | --- | --- | --- | --- |
| rs10420434 | 19 | 45451190 | 19q13.32 | A | G | 6.53×10^-9^ | 4.06×10^-9^ | 0 | *APOC4-APOC2* |
| rs1081105 | 19 | 45412955 | 19q13.32 | C | A | 1.16×10^-232^ | 1.16×10^-231^ | 304 | *APOE* |
| rs112019714 | 19 | 45404857 | 19q13.32 | C | T | 2.02×10^-225^ | 1.72×10^-224^ | 0 | *TOMM40* |
| rs116881820 | 19 | 45397952 | 19q13.32 | C | T | 1.28×10^-206^ | 9.21×10^-206^ | 0 | *TOMM40* |
| rs204470 | 19 | 45488985 | 19q13.32 | A | G | 4.53×10^-8^ | 4.28×10^-8^ | 0 | *CLPTM1* |
| rs204477 | 19 | 45482201 | 19q13.32 | A | G | 5.14×10^-9^ | 4.97×10^-9^ | 0 | *CLPTM1* |
| rs204912 | 19 | 45466207 | 19q13.32 | A | G | 8.78×10^-10^ | 8.26×10^-10^ | 0 | *CLPTM1* |
| rs204913 | 19 | 45466238 | 19q13.32 | T | C | 2.33×10^-9^ | 2.09×10^-9^ | 0 | *CLPTM1* |
| rs204914 | 19 | 45466335 | 19q13.32 | T | C | 2.22×10^-9^ | 1.95×10^-9^ | 0 | *CLPTM1* |
| rs283810 | 19 | 45388241 | 19q13.32 | G | T | 5.44×10^-24^ | 8.54×10^-24^ | 0 | *PVRL2* |
| rs34224078 | 19 | 45383115 | 19q13.32 | G | A | 7.52×10^-15^ | 1.24×10^-14^ | 0 | *PVRL2* |
| rs426555 | 19 | 45366275 | 19q13.32 | T | C | 7.01×10^-24^ | 1.88×10^-23^ | 0 | *PVRL2* |
| rs7251911 | 19 | 45582402 | 19q13.32 | G | C | 1.85×10^-10^ | 8.85×10^-11^ | 0 | *CTB-179K24.3* |
| SNP: single nucleotide polymorphism; A1: effect allele; A2: other allele; *P*_+_: *P-*value for positive direction; *P*_-_: *P-*value for negative direction; *P*_meta_: *P-*value for cross-trait meta-analysis. DIST: Distance to the nearest gene. AD: Alzheimer disease. PTSD: Post-traumatic stress disorder. SNPs which are locating in the gene body or 1kb up- or down-stream of transcription start site or transcription end site have 0. | | | | | | | | | |

**Supplementary Table S1.** Genome-wide significant genes by genome-wide cross-trait analysis associated with AD and PTSD.

**Supplementary Table S2.** Genome-wide significant genes by genome-wide cross-trait analysis associated with AD and ANX.

| **SNP** | **chromosome** | **position** | **Chromosmoe**  **Loci** | **A1** | **A2** | ***P_CPASSOC_*** | ***P_ASSET_*** | **DIST** | **Genes within clumping region** |
| --- | --- | --- | --- | --- | --- | --- | --- | --- | --- |
| rs10182292 | 2 | 127869462 | 2q14.3 | A | G | 1.18×10^-14^ | 1.64×10^-12^ | 4530 | *BIN1* |
| rs7575209 | 2 | 127884123 | 2q14.3 | C | A | 1.66×10^-14^ | 3.69×10^-17^ | 19191 | *BIN1* |
| rs7594230 | 2 | 127873568 | 2q14.3 | A | G | 1.52×10^-14^ | 9.56×10^-13^ | 8636 | *BIN1* |
| rs9268422 | 6 | 32343873 | - | A | G | 4.26×10^-9^ | 2.21×10^-9^ | - | *-* |
| rs12225368 | 11 | 59922815 | - | C | A | 1.74×10^-9^ | 7.85×10^-11^ | 4344 | *AP001257.1* |
| rs148303016 | 19 | 45383830 | 19q13.32 | T | C | 3.96×10^-18^ | 6.26×10^-15^ | 0 | *PVRL2* |
| rs16979933 | 20 | 55020557 | 20q13.31 | C | T | 4.30×10^-8^ | 4.35×10^-8^ | 0 | *CASS4* |
| SNP: single nucleotide polymorphism; A1: effect allele; A2: other allele; *P*_+_: *P-*value for positive direction; *P*_-_: *P-*value for negative direction; *P*_meta_: *P-*value for cross-trait meta-analysis. DIST: Distance to the nearest gene. AD: Alzheimer disease. ANX: Anxiety disorders. SNPs which are locating in the gene body or 1kb up- or down-stream of transcription start site or transcription end site have 0. | | | | | | | | | |

**Supplementary Table S3.** Genome-wide significant genes by genome-wide cross-trait analysis associated with AD and MDD.

| **SNP** | **chromosome** | **position** | **Chromosmoe**  **Loci** | **A1** | **A2** | ***P_CPASSOC_*** | ***P_ASSET_*** | **DIST** | **Genes within clumping region** |
| --- | --- | --- | --- | --- | --- | --- | --- | --- | --- |
| rs10200967 | 2 | 127841769 | 2q14.3 | C | T | 7.61×10^-10^ | 4.17×10^-10^ | 0 | *BIN1* |
| rs1020004 | 7 | 12255778 | 7p21.3 | C | T | 8.31×10^-10^ | 9.94×10^-10^ | 0 | *TMEM106B* |
| rs1060700 | 7 | 12275818 | 7p21.3 | G | A | 1.39×10^-15^ | 1.10×10^-15^ | 0 | *TMEM106B* |
| rs12666249 | 7 | 12260546 | 7p21.3 | T | C | 2.97×10^-10^ | 3.90×10^-10^ | 0 | *TMEM106B* |
| rs1468804 | 7 | 12275508 | 7p21.3 | C | T | 2.28×10^-15^ | 1.55×10^-15^ | 0 | *TMEM106B* |
| rs14978 | 7 | 12273152 | 7p21.3 | G | A | 2.37×10^-10^ | 3.42×10^-10^ | 0 | *TMEM106B* |
| rs1548884 | 7 | 12279761 | 7p21.3 | C | A | 7.94×10^-16^ | 6.98×10^-16^ | 0 | *TMEM106B* |
| rs17149894 | 7 | 12254939 | 7p21.3 | T | C | 1.08×10^-9^ | 1.36×10^-9^ | 0 | *TMEM106B* |
| rs1990622 | 7 | 12283787 | 7p21.3 | G | A | 1.37×10^-15^ | 1.25×10^-15^ | 793 | *TMEM106B* |
| rs3807865 | 7 | 12250402 | 7p21.3 | A | G | 4.51×10^-15^ | 3.86×10^-15^ | 464 | *TMEM106B* |
| rs3807866 | 7 | 12250378 | 7p21.3 | A | G | 5.87×10^-15^ | 4.99×10^-15^ | 488 | *TMEM106B* |
| rs5011432 | 7 | 12268668 | 7p21.3 | C | A | 2.48×10^-15^ | 1.95×10^-15^ | 0 | *TMEM106B* |
| rs6460900 | 7 | 12253088 | 7p21.3 | G | A | 1.77×10^-15^ | 1.16×10^-15^ | 0 | *TMEM106B* |
| rs6945902 | 7 | 12286409 | 7p21.3 | A | C | 2.32×10^-8^ | 3.39×10^-8^ | 3415 | *TMEM106B* |
| rs6966915 | 7 | 12265988 | 7p21.3 | T | C | 2.00×10^-15^ | 1.39×10^-15^ | 0 | *TMEM106B* |
| rs6969722 | 7 | 12278437 | 7p21.3 | A | G | 2.25×10^-10^ | 3.14×10^-10^ | 0 | *TMEM106B* |
| rs2077815 | 11 | 85672383 | 11q14.2 | G | A | 1.32×10^-11^ | 1.47×10^-11^ | 0 | *PICALM* |
| rs592314 | 11 | 85673124 | 11q14.2 | A | G | 5.37×10^-12^ | 5.45×10^-12^ | 0 | *PICALM* |
| rs618679 | 11 | 85671702 | 11q14.2 | A | C | 3.77×10^-12^ | 3.86×10^-12^ | 0 | *PICALM* |
| rs669556 | 11 | 85781322 | 11q14.2 | C | T | 5.84×10^-12^ | 7.05×10^-12^ | 397 | *PICALM* |
| rs2896209 | 14 | 92936600 | 14q32.12 | A | C | 1.61×10^-9^ | 1.57×10^-9^ | 0 | *SLC24A4* |
| rs4406992 | 14 | 92936620 | 14q32.12 | C | T | 2.64×10^-9^ | 2.65×10^-9^ | 0 | *SLC24A4* |
| rs941648 | 14 | 92931737 | 14q32.12 | G | A | 5.36×10^-9^ | 5.95×10^-9^ | 0 | *SLC24A4* |
| SNP: single nucleotide polymorphism; A1: effect allele; A2: other allele; *P*_+_: *P-*value for positive direction; *P*_-_: *P-*value for negative direction; *P*_meta_: *P-*value for cross-trait meta-analysis. DIST: Distance to the nearest gene. AD: Alzheimer disease. MDD: Major depressive disorder. SNPs which are locating in the gene body or 1kb up- or down-stream of transcription start site or transcription end site have 0. | | | | | | | | | |

**Supplementary Table S4** Genomic risk loci and corresponding top variants identified by FUMA with *P*_ASSET_<5×10^–8^ and *P*_CPASSOC_ <5×10^–8^

| **Lead SNP** | **Genomic risk loci** | **Chromosome loci** | | | **n.sig** | ***P_CPASSOC_*** | ***P_ASSET_*** | **Variant annotation** | **Nearest Gene** |
| --- | --- | --- | --- | --- | --- | --- | --- | --- | --- |
| rs7575209 | 2:127840867-127884123 | 2q14.3 | | | 1 | 1.66×10^-14^ | 3.69×10^-17^ | intergenic | *BIN1* |
| rs10200967 | 2:127840867-127884123 | 2q14.3 | | | 1 | 7.61×10^-10^ | 4.17×10^-10^ | intronic | *BIN1* |
| rs1548884 | 7:12233848-12286409 | 7p21.3 | | | 3 | 7.94×10^-16^ | 6.98×10^-16^ | UTR3 | *TMEM106B* |
| rs12225368 | 11:59856028-60041296 | - | | | 1 | 1.74×10^-9^ | 7.85×10^-11^ | intergenic | *AP001257.1* |
| rs618679 | 11:85652251-85783875 | 11q14.2 | | | 1 | 3.77×10^-12^ | 3.86×10^-12^ | intronic | *PICALM* |
| rs2896209 | 14:92927298-92938415 | 14q32.12 | | | 1 | 1.61×10^-9^ | 1.57×10^-9^ | intronic | *SLC24A4* |
| rs148303016 | 19:45361224-45582402 | 19q13.32 | | | 1 | 3.96×10^-18^ | 6.26×10^-15^ | intronic | *PVRL2* |
| rs426555 | 19:45361224-45582402 | | 19q13.32 | 2 | | 7.01×10^-24^ | 1.88×10^-23^ | intronic | *PVRL2* |
| rs1081105 | 19:45361224-45582402 | | 19q13.32 | 2 | | 1.16×10^-232^ | 1.16×10^-231^ | downstream | *APOE* |
| rs204912 | 19:45361224-45582402 | | 19q13.32 | 1 | | 8.78×10^-10^ | 8.26×10^-10^ | intronic | *CLPTM1* |
| rs7251911 | 19:45361224-45582402 | | - | 1 | | 1.85×10^-10^ | 8.85×10^-11^ | intronic | *CTB-179K24.3* |
| rs16979933 | 20:54983075-55040537 | | 20q13.31 | 1 | | 4.30×10^-8^ | 4.35×10^-8^ | intronic | *CASS4* |
| Genomic risk loci are identified by FUMA merging the LD blocks of independent significant SNPs that are closely located to each other (< 250 kb); chromosome loci represent the chromosomal regions containing genomic risk loci; n.sig represents the number of SNPs in the locus. | | | | | | | | | |
